# Estimates of Genetic Differentiation Measured by F_ST_ Do Not Necessarily Require Large Sample Sizes When Using Many SNP Markers

**DOI:** 10.1371/journal.pone.0042649

**Published:** 2012-08-14

**Authors:** Eva-Maria Willing, Christine Dreyer, Cock van Oosterhout

**Affiliations:** 1 Department of Molecular Biology, Max Planck Institute for Developmental Biology, Tübingen, Germany; 2 School of Environmental Sciences, University of East Anglia, Norwich, United Kingdom; Centre National de la Recherche Scientifique, France

## Abstract

Population genetic studies provide insights into the evolutionary processes that influence the distribution of sequence variants within and among wild populations. F_ST_ is among the most widely used measures for genetic differentiation and plays a central role in ecological and evolutionary genetic studies. It is commonly thought that large sample sizes are required in order to precisely infer F_ST_ and that small sample sizes lead to overestimation of genetic differentiation. Until recently, studies in ecological model organisms incorporated a limited number of genetic markers, but since the emergence of next generation sequencing, the panel size of genetic markers available even in non-reference organisms has rapidly increased. In this study we examine whether a large number of genetic markers can substitute for small sample sizes when estimating F_ST_. We tested the behavior of three different estimators that infer F_ST_ and that are commonly used in population genetic studies. By simulating populations, we assessed the effects of sample size and the number of markers on the various estimates of genetic differentiation. Furthermore, we tested the effect of ascertainment bias on these estimates. We show that the population sample size can be significantly reduced (as small as *n* = 4–6) when using an appropriate estimator and a large number of bi-allelic genetic markers (*k*>1,000). Therefore, conservation genetic studies can now obtain almost the same statistical power as studies performed on model organisms using markers developed with next-generation sequencing.

## Introduction

Studies on wild populations give important insights into population dynamics leading to genetic differentiation. One important goal of population genetic studies is to estimate the amount of genetic differentiation among populations in order to draw conclusions on the demographic history. A common measure for the degree of genetic differentiation is the fixation index F_ST_, first defined by Wright (1951).

Until recently, most studies on wild population of non-reference species used moderately large numbers of samples per population (>20), but only a small number of genetic markers (<20), preferentially microsatellites, for which more than two alleles can often be distinguished. Studies on human populations were among the first using thousands of markers, with single nucleotide polymorphisms (SNPs) as markers of choice. SNPs are typically the most abundant sequence variants in genomes. Their distribution throughout the entire genome at high density, combined with well-established models for handling mutation rates and error rates, and inexpensive methods for high throughput genotyping make them appealing for population genetic studies [1]. However, SNP assays are often designed using small panels incorporating only a fraction of populations and individuals that are later genotyped for these SNPs. Consequently, common polymorphisms are more likely detected than rare variants skewing minor allele frequencies (MAF) to higher values [2]. Additionally, because individual SNP assays are expensive to develop, studies on non-reference organisms, and particularly those on wild populations, are relatively rare [2], [3], [4], [5]. New methods incorporating next generation sequencing make it now possible to develop thousands of SNP assays with less bias and at a fraction of previous costs, also in non-reference organisms [6]. Genome-wide datasets provide not only the potential to infer genetic differentiation with higher precision, but also make it possible to detect signatures of selection using empirical F_ST_ outlier methods [7], [8]. It is commonly believed that large sample sizes (*n*>20) are required to yield reliable estimates of differentiation [9], [10], [11], [12]. However, the question arises whether the large increase in the number of available genetic markers reduces the required sample sizes in order to get reliable estimates of F_ST_. Reducing the sample size per population would make it possible to analyze a larger number of different populations at the same cost, and it offers an important advantage in conservation genetic studies on rare organisms. Furthermore, understanding the statistics of different F_ST_ estimators is especially important in the context of detecting regions under selection.

In this study we used simulations to examine whether the estimation of genetic differentiation measured by F_ST_ becomes inflated with small sample sizes. We concentrated on three different estimators. The first one was proposed by Wright (1951), which by definition lies between zero (no genetic differentiation) and one (population have gone to fixation for different alleles). However, Wright assumed infinite sample sizes in his definition, but population size is finite in real datasets. The absence of negative F_ST_ values in Wright's (1951) definition can lead to an overestimation of F_ST_, particularly when the populations are only weakly or not differentiated. Cockerham and Weir (1984) proposed an unbiased estimator that can also have negative F_ST_ estimates and that has been widely used [9]. Therefore, a large number of different simulation studies have been performed in order to compared this estimator to a number of other proposed estimators [10], [13] (see also [12] and citations therein) showing that the different estimators perform quite similarly and give nearly identical values given that sample sizes are large (n>100). Recently, Reich and colleagues [14] proposed a new unbiased estimator for bi-allelic SNP data applicable to very small sample sizes (*n* = 4–6), and the behavior of this new estimator will be evaluated as well. Here we compare all three estimators for their performance on the same bi-allelic data set. We addressed the following four questions. First, what is the effect of small sample size on the type I error rate, i.e. falsely detecting genetic differentiation in a panmictic population? Second, does a small sample size result in an overestimation of the F_ST_ in cases where populations are genetically differentiated? Third, if estimates are imprecise due to small sample sizes, does precision increase with the number of loci genotyped? Fourth, what is the effect of different allele frequency distributions on the F_ST_ statistics, in particular, does a relative excess of common or rare alleles lead to a bias in F_ST_ estimates?

## Results

After *t* generations of random mating, we estimated F_ST_ on the complete simulated dataset comprising two populations with 1,000 individuals each that were genotyped at 21,000 loci (see Material and Methods). All three estimators gave nearly identical values on the complete dataset at all levels of genetic differentiation. However, all estimators tended to give a slightly higher value than the theoretically expected F_ST_ (Table 1). The reason is that there is variance in the offspring number around the Binomial distribution, which slightly inflates the observed F_ST_ compared to the theoretically expected value. In simulated data, the occasional extreme value in reproductive variance (i.e. a family producing 5 or 6 offspring instead of the mean expected number 2) will cause additional drift and differentiation over and beyond that expected from the theoretical distribution. Because of the absorption state of SNP loci (an allele getting fixed or lost from the gene pool), such random chance events are not completely offset by an overly equal reproductive distribution (i.e. runs in which too many families with exactly 2 offspring). As a consequence, the simulated F_ST_, like a ratchet, tends to increase slightly faster than the theoretically expected value.

**Table 1:** Estimated F_ST_ values on complete dataset.

expected F_ST_	# generations	*F_ST_^W^*	*F_ST_^W&C^*	*F_ST_^R^*
0	0	0	−5.00E-04	−5.00E-04
0.0104	21	0.0107	0.0102	0.0102
0.0503	103	0.0542	0.0546	0.0547
0.1003	211	0.1073	0.1097	0.1096
0.2	447	0.2022	0.2068	0.2068
0.4001	1022	0.4134	0.4024	0.4023

### Estimates on SNP set with uniform allele frequency distribution

**Figure 1:**
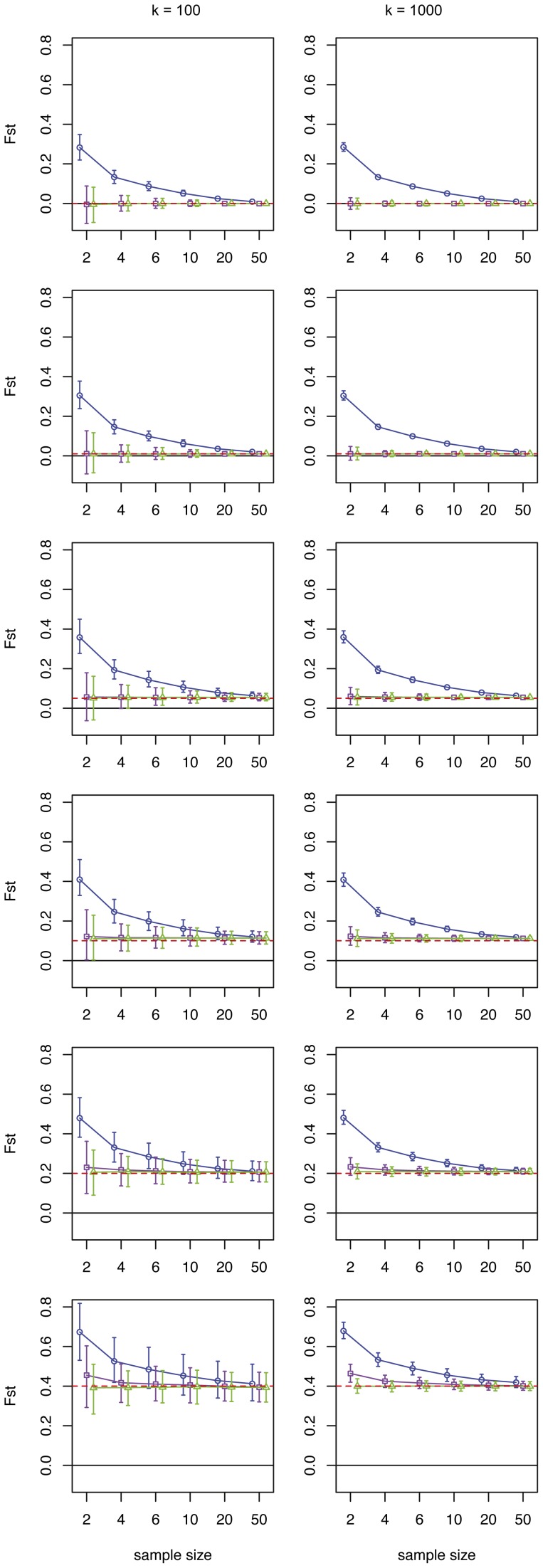
Effect of increasing sample sizes. Results are shown for the simulations where allele frequencies were equally distributed from 0.05 to 0.95. The number of markers was fixed at k = 100 (left column) and k = 1,000 (right column). Each row contains a different level of genetic differentiation (F_ST_ = 0, 0.01, 0.05, 0.1, 0.2, 0.4). The results (average F_ST_ and 95% CI) of each estimator are depicted in the different graphs: F_ST_^W^ (blue circles), F_ST_^C&W^ (purple squares) and F_ST_^R^ (green triangles). The dashed red line indicates the actual F_ST_ for the simulated population.

**Figure 2:**
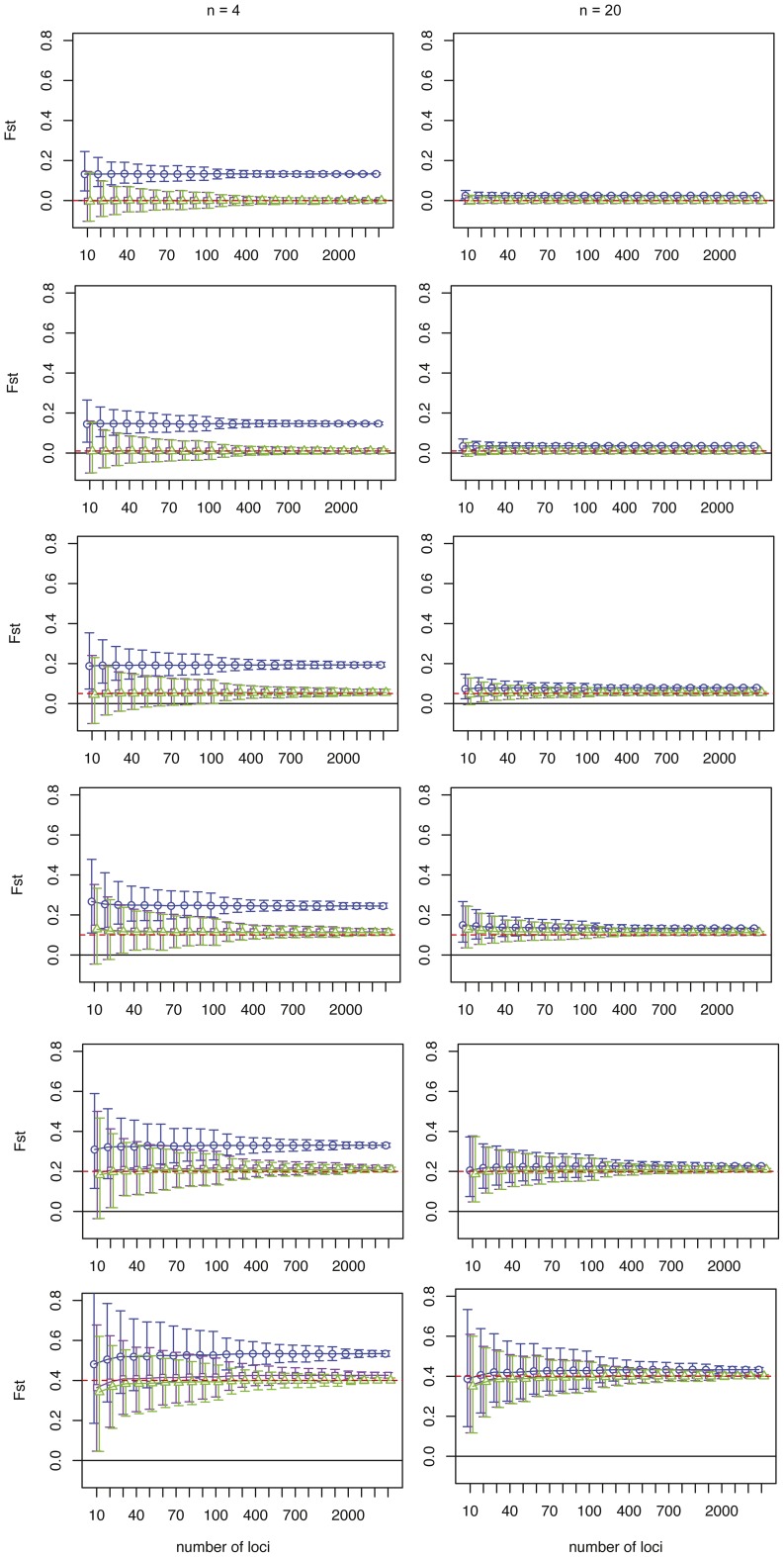
Effect of increasing the number of markers. Results are shown for the simulations where allele frequencies were equally distributed from 0.05 to 0.95. The number of individuals was fixed at n = 4 (left column) and n = 20 (right column). Each row contains (like in Figure 1) a different level of genetic differentiation (F_ST_ = 0, 0.01, 0.05, 0.1, 0.2, 0.4). The results (average F_ST_ and 95% CI) of each estimator are depicted in the different graphs: F_ST_^W^ (blue circles), F_ST_^C&W^ (purple squares) and F_ST_^R^ (green triangles). The dashed red line indicates the actual F_ST_ for the simulated population.

We tested the influence of increasing the sample size on the estimate by taking 2, 4, 6, 10, 20 and 50 individuals from each population at different levels of genetic differentiation (*F_ST_* = 0, 0.01, 0.05, 0.1, 0.2, 0.4). Figure 1 shows an example were the number of loci is fixed at *k* = 100 and *k* = 1,000 at varying sample sizes. Figure 2 depicts an example were the number of individuals is fixed at *n* = 4 and *n* = 20 at increasing number of loci genotyped. Since combining all the different parameters (sample size, number of loci and level of genetic differentiation) resulted in a large number of estimates, we chose these four combinations in order to illustrate our general findings (all estimates can be downloaded as supplementary file). *F_ST_^W^* severely overestimated genetic differentiation in small sample sizes (*n* = 2–6) (e.g. Figure 1). Moreover, since this estimator cannot have negative values, the 95% CIs excluded zero implying significant genetic differentiation even if there is none. Also with moderate sample sizes (*n* = 10–50), *F_ST_^W^* slightly overestimates the level of genetic differentiation. Since these observations were consistent for all datasets, we will in the following concentrate on the behavior of the two other estimators.

The estimators *F_ST_^W&C^* and *F_ST_^R^* gave on average similar, fairly good estimates at all sample sizes (Figure 1). Importantly, both estimators did not indicate genetic differentiation when there was none (Figure 1). However, if genetic differentiation was moderate or large (F_ST_≥0.1) the F_ST_ *^W&C^* estimator tended to slightly overestimate genetic differentiation with small sample sizes (*n*≤6), whereas the estimator F_ST_ *^R^* showed the same average estimate irrespective of samples size. Though, with increasing sample sizes the size of 95% CIs decreased. The 95% CIs were large and included zero at low genetic differentiation (F_ST_<0.05), when using a small sample size (*n* = 2–6) and a moderate number of loci (Figure 1, *k* = 100). Increasing the number of loci had no impact on the average estimate of F_ST_ (Figure 1, k = 1,000; Figure 2), but it did significantly reduce the 95% CIs. This effect was similar to the reduction in 95% CI caused by increasing the sample sizes (Figure 2). With 50 individuals per population and 1,000 loci one can detect genetic differentiation as small as 0.001 (average = 0.0011, 95% CI = [1.12E-04, 0.0022], see Tables S1, S2 and S3). Genetic differentiation as small as 0.01 can already be detected with *n* = 4 and *k* = 3000 (average = 0.0102, 95% CI = [0.0014, 0.0212], see \ Tables S1, S2 and S3).

### Influence of unequal sample sizes

Next, we considered the impact of unequal sample sizes on the F_ST_ estimates. For this we kept the sample size taken from population 1 fixed at *n_1_* = 4 and varied the sample size taken from population 2. At low genetic differentiation (F_ST_≤0.05) differences between sample sizes did not have an impact on the average estimates of F_ST_ of either estimator (F_ST_ *^W&C^* and F_ST_ *^R^*). If genetic differentiation was moderate to high (F_ST_≥0.1), the F_ST_ *^W&C^* overestimated genetic differentiation when the sample size of population 2 was small (*n_2_*≤6, see Figure S1), but it gave an underestimation in cases where the sample taken from population 2 was large (*n_2_* = 50, see Figure S1). The F_ST_ *^R^* estimator gave on average the same estimate of F_ST_ independently of the differences between sample sizes. Furthermore the estimates were always very close to the expected level of genetic differentiation (see Figure S1). Therefore, we would recommend F_ST_^R^, if sample sizes differ among the populations analyzed. However, the magnitude of the 95% CI did not decrease with increasing the sample size in only one population of either estimator leading to the conclusion that the accuracy of an estimate depends on the smaller sample size taken.

### Estimates on SNP sets with different allele frequency distributions

In order to test the influence of biases of allele frequency distribution in the analysed marker set, we generated two datasets with 10,000 loci each, where in one set MAF>0.25 and in the other set MAF≤0.25. The simulations show that a shift towards common polymorphisms in the marker set leads to overestimation of genetic differentiation, whereas a shift towards rare polymorphisms leads to underestimation. These erroneous estimates were observed in both the *F_ST_^R^* estimator as well as the F_ST_ *^W&C^* estimator, and they could neither be compensated by increasing the sample size nor by increasing the number of loci genotyped (see Table 2 and Figures S2 and S3). This suggests there will be a systematic over- or underestimation of genetic differentiation, if the allele frequency distribution inferred from the samples does not reflect the underlying distribution of the total population (e.g. if there is an ascertainment bias or unrepresentative sampling). Therefore, using genetic markers developed in a panel not containing all populations analyzed can lead to wrong estimates of genetic differentiation. A bias towards common polymorphisms in the marker set will lead to an overestimation of genetic differentiation, and *visa versa*. The practical implication is that when the marker loci in the panel are developed based on the pre-screening of a small number of individuals (e.g. n = 4), this will lead to ascertainment bias and an overestimation of genetic differentiation.

**Table 2:** Estimated F_ST_ values changed allele frequency distributions, n = 50, k = 1000.

	*MAF*≤0.25		*MAF>0.25*	
expected F_ST_	*F_ST_^W&C^*	*F_ST_^R^*	*F_ST_^W&C^*	*F_ST_^R^*
0	−5.10E-04	−5.09E-04	−5.13E-04	−5.13E-04
0.0104	0.0100	0.0100	0.0103	0.0102
0.0503	0.0502	0.0502	0.0572	0.0572
0.1003	0.0962	0.0961	0.1163	0.1161
0.2	0.1597	0.1594	0.2266	0.2260
0.4001	0.2389	0.2384	0.4649	0.4628

## Discussion

Although the statistical behavior of different F_ST_ estimators has been analyzed before, this study is the first to evaluate different F_ST_ estimators in population genetic studies of thousands of loci and very small sample sizes. This is a timely matter, given that Next Generation Sequencing (NGS) methods have revolutionized the field of marker development. Furthermore, we have included a new estimator employed by Reich et al. (2009). Our simulations show that even when sample sizes are small (*n* = 2, 4, 6), accurate and unbiased estimates of F_ST_ can still be obtained when a large number of bi-allelic markers such as SNPs are used, as long as the appropriate F_ST_ estimator is chosen. The original F_ST_^W^ estimator severely overestimates the level of genetic differentiation when using small sample sizes. Since this estimator cannot have negative values, these results were expected for values of F_ST_<0.5, because overestimates that are higher than 0.5 over the actual F_ST_ cannot be compensated by a negative value at another locus. The two other estimators we tested showed similar performance on large sample sizes (*n*≥20), and consistent with a previous study [12], the F_ST_^W&C^ overestimates F_ST_ when analyzing small samples. Importantly, the estimator proposed by Reich et al. (2009) showed better performance in cases where sample sizes were small (*n*≤6). Our simulations suggest that genetic differentiation is not falsely detected due to small sample sizes when using this unbiased estimator. Furthermore, we showed that increasing the number of genetic markers has no impact on the mean F_ST_ estimates, but that it considerably reduces the 95% CIs. Although this is not unexpected, given that increasing the number of loci decreases the coefficient of variation of the estimates, we quantify this effect here for the first time. Finally, the precision of a pairwise estimate depends on the smaller sample size taken from one of the populations and not on the number of loci.

A previous study suggested that increasing the sample size might be more beneficial than increasing the number of markers genotyped [11]. However, that study tested a rather small number of SNPs (k<100). Our study suggests that using a large number of markers (>500) increases significantly the power of detecting genetic differentiation even if using a small sample size. For example, pairwise genetic differentiation as small as 0.01 can be detected by taking a sample of only four individuals from each population when genotyped at 3,000 loci. This finding has important implications for studies on endangered species or those with small population size. By developing markers using next generation sequencing tools, conservation genetic studies can obtain the same statistical power in some of their population genetic analysis as studies performed on model organisms. However, testing marker sets with different allele frequencies (MAF≤0.25 and MAF>0.25) has shown that deviation from the true underlying distribution of allele frequencies has a severe effect on the estimation of F_ST_ rather than the sample size taken. A bias towards common polymorphisms in the marker set leads to overestimation of genetic differentiation, whereas, *visa versa*, F_ST_ becomes underestimated when there is a bias towards rare polymorphisms. The practical implication is that if the panel is developed based on the screening of a small number of individuals, this will lead to ascertainment bias and an overestimation of genetic differentiation of the study populations.

Theoretically, precise estimates of genetic differentiation can be obtained based on very small sample sizes of *random* individuals. However, in practice, random sampling cannot always be achieved in nature. Estimates on extremely small samples, as suggested here, might be severely affected by non-random sampling, e.g. due to population structuring. Furthermore, our simulated dataset contained only fully unlinked markers. If subsets of markers are linked they represent only one informative marker. Hence, the number of fully unlinked markers depends on the genome size of the organism analyzed. For the same reason, detecting regions under selection with F_ST_ outlier methods require larger sample sizes (n>10). In such cases, F_ST_ estimates are based on small sliding windows in which markers are (partially) linked, and hence, they represent a smaller number of independent loci. Baird and colleagues (2008) proposed a method that has been proven particularly useful to develop a large number of genetic markers in non-reference organisms with less ascertainment bias [15], [16]. Using multiplex strategies, samples taken from different populations in the wild can now be sequenced and genotyped in one lane of Illumina GAIIX sequencer [17]. Therefore, it is now possible to analyze genetic differentiation from a large number of populations at low cost. Our simulations have shown that the cost of these analyses can be even reduced further by using only a small number of individuals per population.

## Methods

### Data generation

We simulated an ancestral population with 1,000 individuals (50% males and 50% females, sex assigned randomly) and 21,000 bi-allelic loci. The genotypes at the loci were generated by randomly drawing from eight allele frequency classes (0.1, 0.2, …., 0.9). The two starting populations consisted of the same 1,000 individuals, which were genotyped at 10,000 loci randomly taken from the 21,000 loci of the ancestral population. We assumed an isolated island model (i.e. no migration between the two populations after separation). Genetic drift was simulated for a certain number (*t*) of generations according to the Wright-Fisher model without mutations (which is appropriate for SNPs, which arise at much lower rates than microsatellite variants). Consequently, the population sizes were kept constant, the generations were non-overlapping and the frequencies in the next generation were a binomial random sample based on the frequencies in the current generation. Random dioecious mating was simulated by randomly drawing one female and one male with replacement. Thus all males and females were equally likely to be chosen and could mate multiple times, and the draw was independent of the number of times an individual has been chosen before. The two individuals drawn became the parents of one member of the next generation. Since we assumed all loci to be completely unlinked, we simulated gametogenesis by simply selecting at random one allele from each parent. This process was repeated until all members of the next generation have been created. We simulated different degrees of genetic differentiation (F_ST_ = 0, 0.01, 0.05, 0.1, 0.2). The number *t* of generations was determined by 
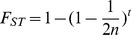
 with *n* equals the number of mating individuals (effective population size, N_e_ = 1,000) and *t* equals the number of generations needed to achieve the required amount of differentiation [9], [11].

### Estimators tested

F_ST_ was introduced by Wright (1951) as a measure of correlation of gene frequencies und suggested the first and simplest estimator, *F_ST_^W^*. For one allele at locus *k*,
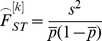
where
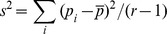
is the observed variance of allele frequencies *p_i_* among the sampled populations *i* (*i* = 1, …, *r*) and 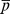 is the mean allele frequency over all populations. The estimate of *F_ST_^W^* for multiple loci is calculated by taking the mean across *k* loci.
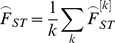
This estimator has a theoretical range between zero and one and is known to overestimate the level of genetic differentiation especially at low values [18].

The second estimator tested, *F_ST_^W&C^*, preserves Wright's definition of F_ST_ in terms of correlation of gene frequencies and is the most widely used estimators (cited approx. 7,000 times, source: Web-of-Science) [9], [12]. It was proposed by Weir & Cockerham (1984) [18], who showed that it provides a nearly unbiased estimate of F_ST_ at moderate population sample size (*n* = 15, 20 and 25) and small number of loci (*k* = 10). The estimates can also have negative values which do not have a biological meaning [19], but they can compensate for overestimates especially at low levels of genetic differentiation. At a single locus *k*, *F_ST_^W&C^* is defined as
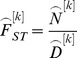
where
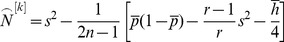


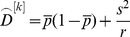
Here, *s^2^* is the observed variance of allele frequencies, *n* is the number of individuals per population, 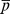 is the mean allele frequency over all populations, *r* is the number of sampled populations and 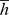 is the mean observed heterozygosity. The overall estimate from all *k* loci is derived by
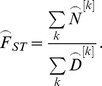



Recently, Reich and colleagues (2009) proposed a new unbiased estimator, *F_ST_^R^*, for bi-allelic loci and pairwise population comparison. In their study they used a very high number of loci, but small sample sizes per population. Therefore, we decided to test this estimator as well. Again *F_ST_^R^* is calculated as follows
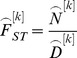


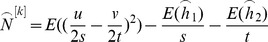


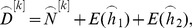
where *u* is the allele count for population 1, *v* is the allele count for population 2, *t* and *s* are the total number of individuals for population 1 and 2, respectively [14]. The parameter 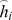 is an unbiased estimate of the expected heterozygosity. An estimate over many loci is given by
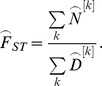



### Statistical analysis

After *t* generations of random mating among 1,000 individuals, 10,000 of the 21,000 loci were randomly chosen to test the *F_ST_* estimates. In order to test the influence of ascertainment bias in marker design, we generated three different datasets. The first set contained loci with equally distributed allele frequencies, the second set contained only loci with minimum allele frequency MAF>0.25, because SNP marker sets are often biased in the direction of more common polymorphisms. However, we also generated a dataset of the other extreme containing only markers with MAF≤0.25.

We used sample sizes of 2, 4, 6, 10, 20 and 50 individuals. For each sample size we sampled 10, 20, …, 100, 200, …, 1000, 2000, …, 5000 loci. For each number of individuals and genotyped loci we sampled from each population 1,000 times. We took the average F_ST_ estimate and derived the 95% confidence interval. We used a custom Java program to perform the simulations and estimations of F_ST_ that will be made available upon request.

## Supporting Information

Figure S1 **Effect of increasing sample sizes and increasing marker numbers with uniform allele frequency distribution.** Results are shown for the simulations where allele frequencies were equally distributed from 0.05 to 0.95. The number of markers was fixed at k = 100 (left column) and k = 1,000 (left middle column). The number of individuals was fixed at n = 4 (right middle column) and n = 20 (right column). Each row contains a different level of genetic differentiation (F_ST_ = 0, 0.01, 0.05, 0.1, 0.2, 0.4). The results (average F_ST_ and 95% CI) of each estimator are depicted in the different graphs: F_ST_^W^ (blue circles), F_ST_^C&W^ (purple squares) and F_ST_^R^ (green triangles). The dashed red line indicates the actual F_ST_ for the simulated population.
(PDF)

Figure S2 **Effect of increasing sample sizes and increasing marker numbers for MAF>0.25.** Results are shown for the simulations where allele frequencies were equally distributed from 0.05 to 0.95. The number of markers was fixed at k = 100 (left column) and k = 1,000 (left middle column). The number of individuals was fixed at n = 4 (right middle column) and n = 20 (right column). Each row contains a different level of genetic differentiation (F_ST_ = 0, 0.01, 0.05, 0.1, 0.2, 0.4). The results (average F_ST_ and 95% CI) of each estimator are depicted in the different graphs: F_ST_^W^ (blue circles), F_ST_^C&W^ (purple squares) and F_ST_^R^ (green triangles). The dashed red line indicates the actual F_ST_ for the simulated population.
(PDF)

Figure S3 **Effect of increasing sample sizes and increasing marker numbers for MAF<0.25.** Results are shown for the simulations where allele frequencies were equally distributed from 0.05 to 0.95. The number of markers was fixed at k = 100 (left column) and k = 1,000 (left middle column). The number of individuals was fixed at n = 4 (right middle column) and n = 20 (right column). Each row contains a different level of genetic differentiation (F_ST_ = 0, 0.01, 0.05, 0.1, 0.2, 0.4). The results (average F_ST_ and 95% CI) of each estimator are depicted in the different graphs: F_ST_^W^ (blue circles), F_ST_^C&W^ (purple squares) and F_ST_^R^ (green triangles). The dashed red line indicates the actual F_ST_ for the simulated population.
(PDF)

Table S1 **F_ST_ estimates according to Wright (1951).**
(XLSX)

Table S2 **F_ST_ estimates according to Weir & Cockerham (1984).**
(XLSX)

Table S3 **F_ST_ estimates according to Reich et al. (2009).**
(XLSX)
