# Supplementary figures and images for: Estimates of Genetic Differentiation Measured by FST Do Not Necessarily Require Large Sample Sizes When Using Many SNP Markers
Source: PLoS One. 2012 Aug 14;7(8):e42649. doi: 10.1371/journal.pone.0042649 (PMC3419229; doi:10.1371/journal.pone.0042649)

**Figure S1: Normal allele frequencies**

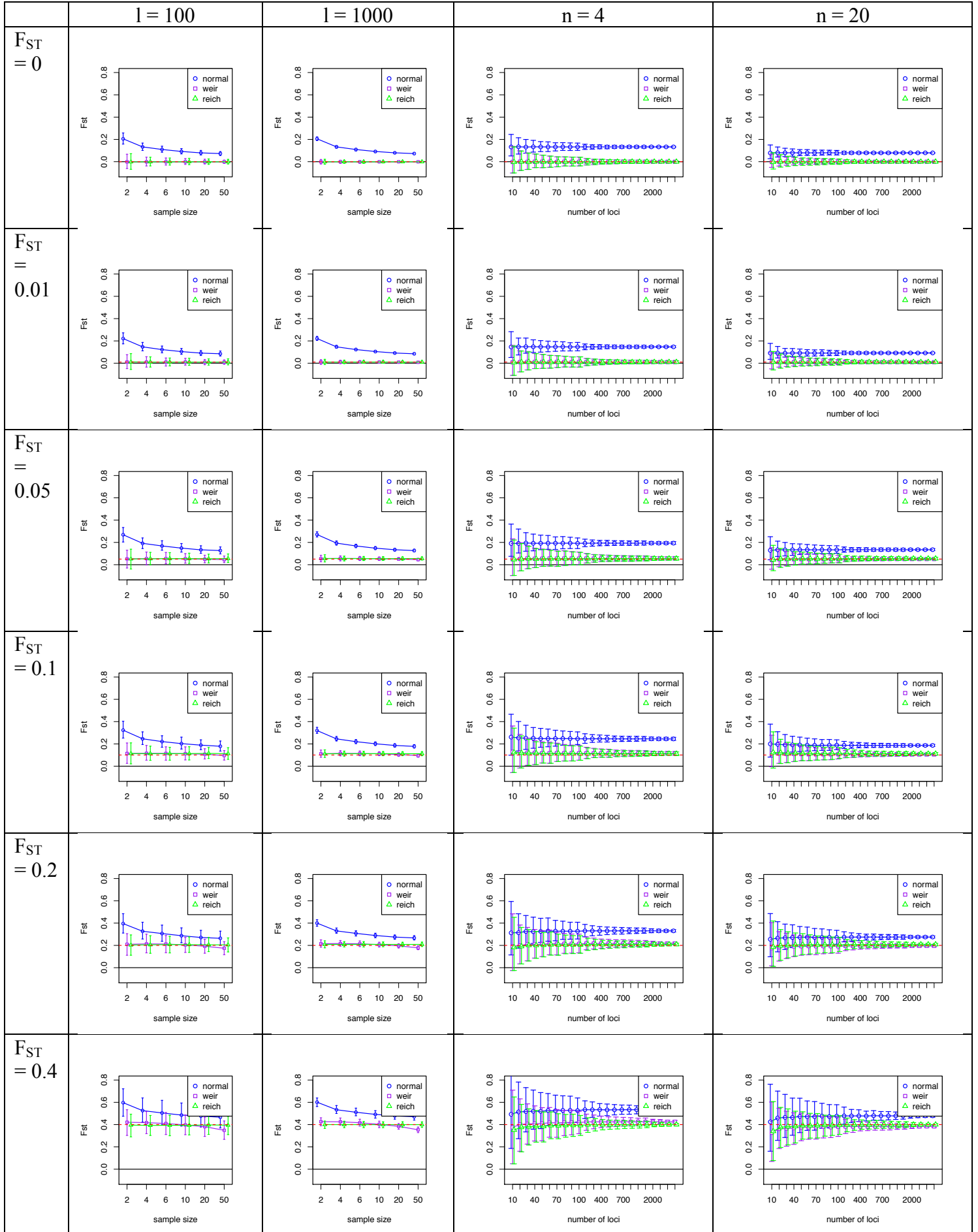

Supplement: Figure S1 — Effect of increasing sample sizes and increasing marker numbers with uniform allele frequency distribution. Results are shown for the simulations where allele frequencies were equally distributed from 0.05 to 0.95. The number of markers was fixed at k = 100 (left column) and k = 1,000 (left middle column). The number of individuals was fixed at n = 4 (right middle column) and n = 20 (right column). Each row contains a different level of genetic differentiation (FST = 0, 0.01, 0.05, 0.1, 0.2, 0.4). The results (average FST and 95% CI) of each estimator are depicted in the different graphs: FST W (blue circles), FST C&W (purple squares) and FST R (green triangles). The dashed red line indicates the actual FST for the simulated population. (PDF) [file pone.0042649.s001.pdf]

**Figure S2: Skewed major allele frequencies (MAF > 0.25)**

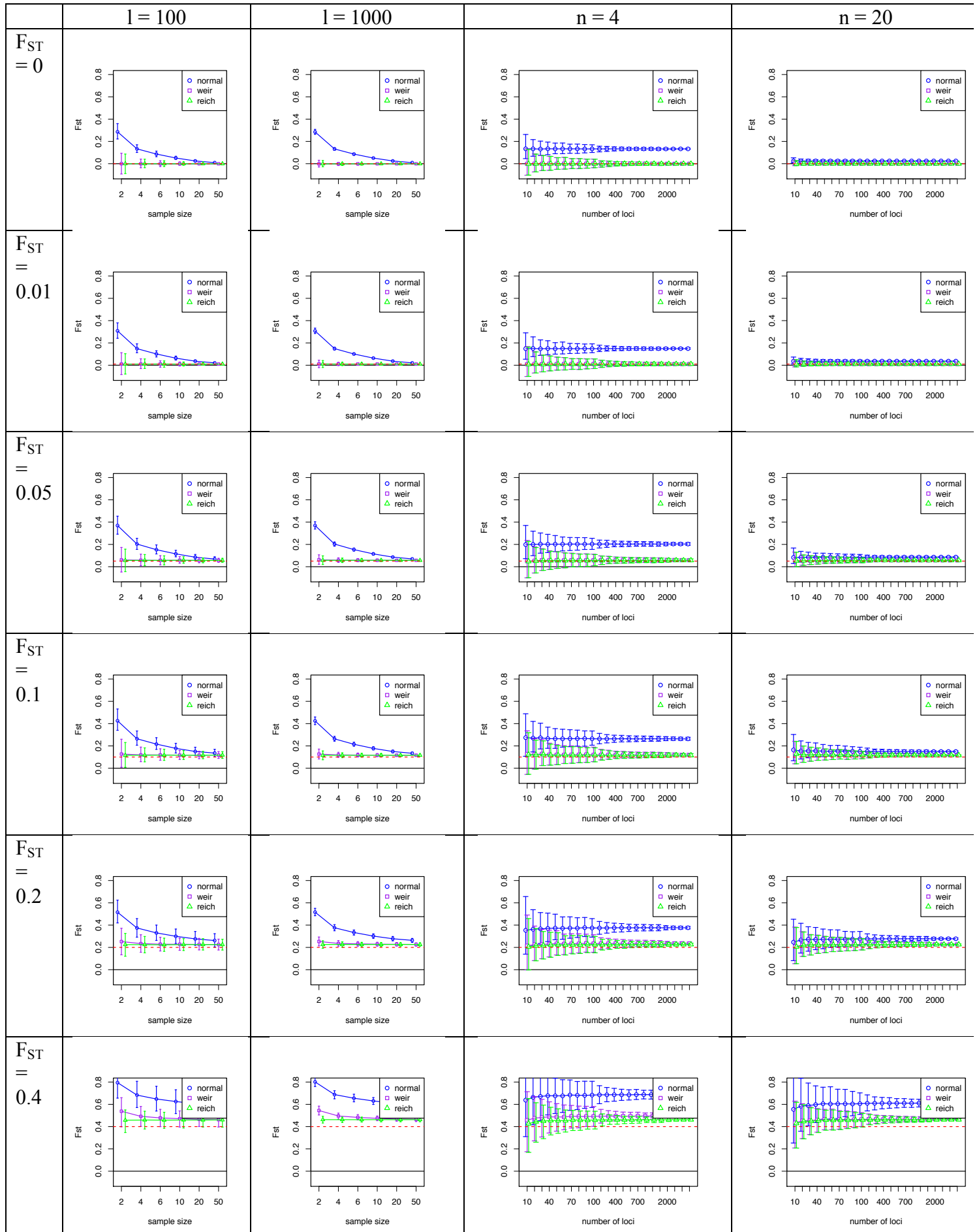

Supplement: Figure S2 — Effect of increasing sample sizes and increasing marker numbers for MAF>0.25. Results are shown for the simulations where allele frequencies were equally distributed from 0.05 to 0.95. The number of markers was fixed at k = 100 (left column) and k = 1,000 (left middle column). The number of individuals was fixed at n = 4 (right middle column) and n = 20 (right column). Each row contains a different level of genetic differentiation (FST = 0, 0.01, 0.05, 0.1, 0.2, 0.4). The results (average FST and 95% CI) of each estimator are depicted in the different graphs: FST W (blue circles), FST C&W (purple squares) and FST R (green triangles). The dashed red line indicates the actual FST for the simulated population. (PDF) [file pone.0042649.s002.pdf]

**Figure S3: Skewed allele frequencies (MAF < 0.25)**

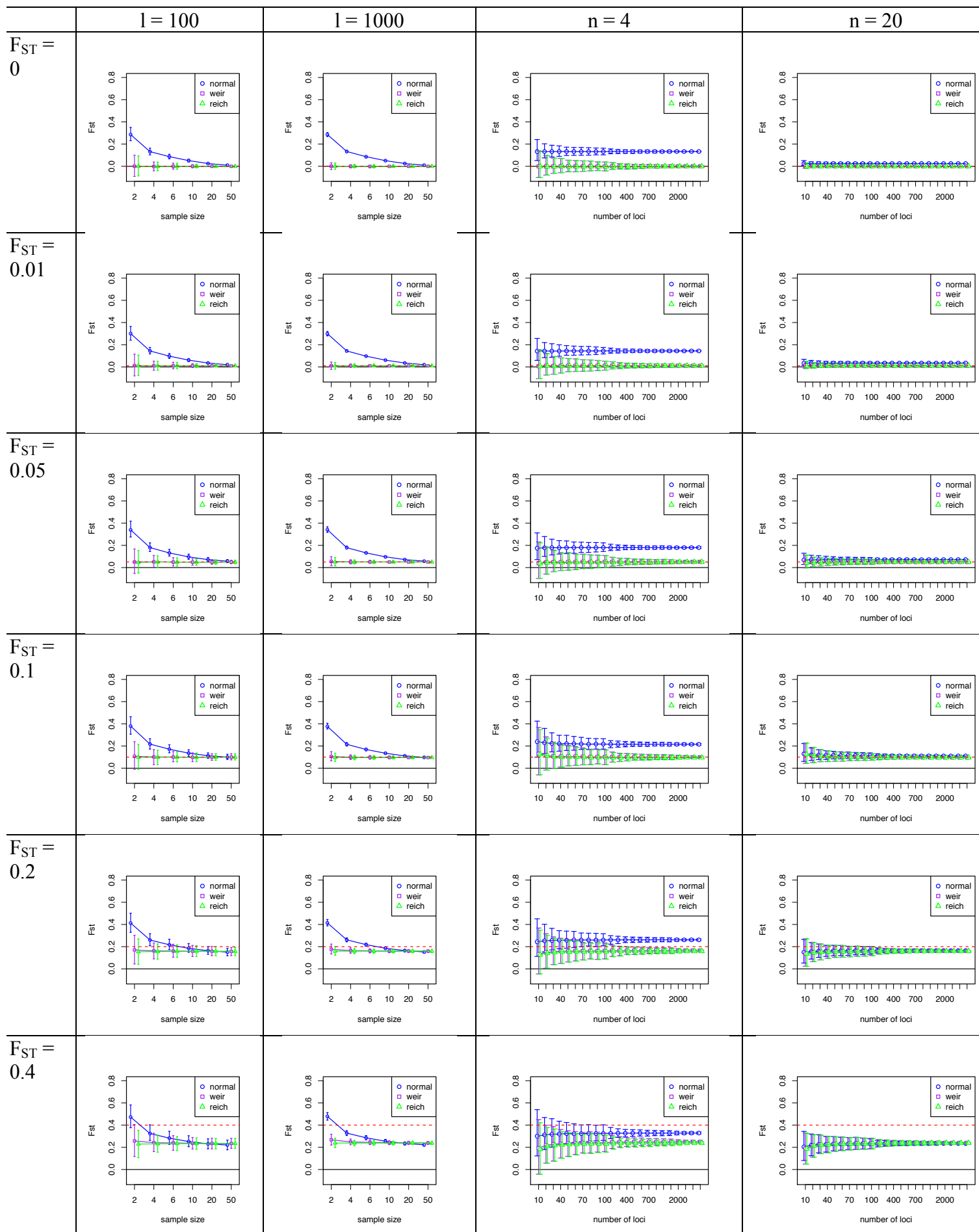

Supplement: Figure S3 — Effect of increasing sample sizes and increasing marker numbers for MAF<0.25. Results are shown for the simulations where allele frequencies were equally distributed from 0.05 to 0.95. The number of markers was fixed at k = 100 (left column) and k = 1,000 (left middle column). The number of individuals was fixed at n = 4 (right middle column) and n = 20 (right column). Each row contains a different level of genetic differentiation (FST = 0, 0.01, 0.05, 0.1, 0.2, 0.4). The results (average FST and 95% CI) of each estimator are depicted in the different graphs: FST W (blue circles), FST C&W (purple squares) and FST R (green triangles). The dashed red line indicates the actual FST for the simulated population. (PDF) [file pone.0042649.s003.pdf]
